# Characterization of a new genotype of avian bornavirus from wild ducks

**DOI:** 10.1186/s12985-014-0197-9

**Published:** 2014-11-19

**Authors:** Jianhua Guo, H L Shivaprasad, Raquel R Rech, Jill J Heatley, Ian Tizard, Susan Payne

**Affiliations:** Department of Veterinary Pathobiology, MS#4456, Texas A&M University, College Station, TX 77843 USA; The California Animal Health and Food Safety Laboratory System - Tulare, University of California, Davis, USA; Department of Small Animal Clinical Sciences, Texas A&M University, College Station, TX 77843 USA

**Keywords:** Avian bornavirus, Genotype, Mallard

## Abstract

**Background:**

Avian bornaviruses (ABV) are a recently described group of intranuclear negative-stranded RNA viruses (Order *Mononegavirales*, Family *Bornaviridae*). At least 13 different ABV genotypes have been described. One genotype, the Canada goose genotype (ABV-CG), has been isolated from geese and swans and is widely distributed across North America.

**Results:**

We have isolated and characterized a previously undescribed genotype of avian bornavirus from the brains of wild ducks. This new genotype, provisionally designated ABV genotype MALL, was detected in 12 of 83 mallards, and 1 of 8 wood ducks collected at a single location in central Oklahoma. The virus was cultured on primary duck embryo fibroblasts, fragments were cloned, and its genome sequence of 8904 nucleotides determined. This new genotype has 72% nucleotide identity and 83% amino acid identity with the ABV-CG genotype previously shown to be present in geese and swans. Histologic and immunohistochemical examination of the brains and eyes of four positive ducks indicated the presence of virus-infected neurons and glia in their cerebrums and retinas in the absence of inflammation.

**Conclusions:**

More than one genotype of ABV is circulating in North American waterfowl. While the infected ducks were not observed to be suffering from overt disease, based on the immunohistochemistry, we speculate that they may have suffered some visual impairment.

**Electronic supplementary material:**

The online version of this article (doi:10.1186/s12985-014-0197-9) contains supplementary material, which is available to authorized users.

## Introduction

Avian bornaviruses (ABV) are negative sense, single stranded RNA viruses belonging to the Family *Bornaviridae*. Neurologic disease caused by Borna disease virus (BDV) has long been recognized in mammals. BDV may also be able to infect birds, including mallards [1] and ostriches [2]. Avian bornaviruses (ABV) in contrast, were first identified as the causal agents of proventricular dilatation disease (PDD), a lethal neurologic disease of captive parrots in 2008 [3,4]. Subsequent studies in this and other laboratories have demonstrated the occurrence of ABV in wild waterfowl, specifically Canada geese (*Branta canadensis*) [5,6]*,* snow and Ross’s geese (*Chen caerulescens* and *Chen rossii*) [7] and both mute [8] and whooper swans (*Cygnus olor* and *C. buccinator*) [5,6]. ABV has been isolated from Canada goose and swan brain samples obtained from multiple sites in the United States and Canada [6,8]. Genotyping has shown that the goose and swan isolates in North America belong to a distinct Canada goose (ABV-CG) genotype that differs from the ABV genotypes previously characterized in psittacines and passerines [9] as well as from BDV in mammals. The significance of ABV infection in these large waterfowl is unclear. While most birds sampled by us appeared to be healthy, the virus has been detected in the brains of geese and swans suffering from severe neurologic disease on several occasions at different locations in the United States and Canada. In a recent study, Delnatte et al. [10] described a range of clinical and behavioral abnormalities in ABV-infected Canada geese in a flock in Toronto, Canada. These abnormalities ranged from minor behavioral defects to significant clinical signs such as an inability to fly or walk. Other geese developed proventricular impaction in a manner similar to proventricular dilatation disease in psittacines and typical cases resembling PDD have been recorded in Canada geese [11]. Similar neurologic signs of encephalitis and PDD-like lesions associated with ABV infection as demonstrated by RT-PCR and immunohistochemistry have been observed in Canada geese in California (Shivaprasad, Unpublished). Thus ABV cannot be regarded as an innocuous virus although the magnitude of its threat to health and survival in wild birds is unclear. In addition to infection in Anseriformes, ABV-CG has also been detected in a bald eagle (*Haliaeetus leucocephalus*) with lethal encephalitis (This is a species that predates on sick waterfowl) [7].

In order to further clarify the distribution of ABV in North American waterfowl and to determine the diversity of species infected, we have surveyed wild ducks for the presence of this virus. Based upon previous results from mute swans and Canada geese, brains were considered the tissues of choice for ABV detection. Fresh brain samples were therefore obtained from 98 hunter-killed ducks in Central Oklahoma in January 2014.

These brain tissues were screened by reverse transcriptase-PCR for RNA encoding the matrix (M) gene of ABV. PCR products, 350 bp in size, were detected in 13 of these birds and were sequenced in order to determine the viral genotype. Selected brain samples were grown in primary duck embryo fibroblasts and the cultured virus was characterized. The entire genome was sequenced confirming that the virus belongs to a previously undescribed genotype. Histopathologic examination of the brains and eyes of four PCR-positive mallards revealed no significant lesions. However, immunohistochemistry of the brain and eyes from an RT-PCR-positive bird revealed the presence of large numbers of virus-infected cells, in both the cerebrum and in the retina.

## Materials and methods: samples

Eighty three mallards (*Anas platyrhynchos*), 8 wood ducks (*Aix sponsa*), 4 green- winged teal (*Anas crecca*), and 3 pintails (*Anas acuta*), were killed by hunters between 14 and 28 January 2014 at the Deep Fork Arm of Eufaula Lake, McIntosh County, OK. (35° 28'4.85''N: 95° 48'22.99''W). The heads were removed from freshly killed birds, held on ice and shipped to this laboratory. Most were stored at −80°C until tested. Twenty were held at 4°C in order to retain structural integrity for histopathology and immunohistochemistry.

### Sample processing

The frozen heads were permitted to thaw at 4°C for 48 hours. Brain tissue samples were then obtained by syringe extraction of a portion of tissue through a 16 gauge needle inserted into the foramen magnum. These samples were refrozen until tested by reverse transcriptase polymerase chain reaction (RT-PCR). Once RT-PCR results were obtained, brains from all the ABV-positive and two ABV-negative birds were removed, fixed in buffered formalin, processed and examined by conventional histopathology and immunohistochemistry by pathologists unaware of the RT-PCR results.

### Virus culture

A 10% suspension of the forebrain and cerebellum was homogenized in MEM-Earle complete medium containing 10% fetal bovine serum (FBS) and 50⌠g/ml penicillin/streptomycin using VWR® disposable tissue grinders, clarified by centrifugation at 10,000 g for 10 minutes and then passed through a 0.2 micron filter. Five ml of this brain suspension was used to inoculate DEF monolayers in 25-cm^2^ flasks. The inoculated DEFs were incubated at 37°C in an atmosphere of 5% CO_2_ for 24 hours. The cultures were then washed once with phosphate- buffered saline (PBS), replaced with complete minimum essential medium (MEM) supplemented with 2% FBS and incubated for an additional 5–7 days. DEFs were trypsinized (0.25% trypsin) and passaged 4 times.

### RNA purification

RNA was extracted from brain tissue and from infected DEFs using an RNeasy Mini kit (Qiagen) according to the manufacturers instructions. The RNA concentration was determined using a NanoDrop® ND-1000 UV–vis Spectrophotometer.

### cDNA synthesis

First strand cDNA was generated using the Applied Biosystems® High Capacity cDNA Reverse Transcription Kit (Applied Biosystems), using 10 μl RNA (~500 ng) and random primers. The final composition of cDNA reaction mixtures was 2 μl of 10X buffer, 2 μl of 10X random primers, 0.8 μl of 100 mM dNTP mix, 1 μl RNAse inhibitor, and 1 μl reverse transcriptase in a final volume of 20 μl.

### RT-PCR screening

A primer set targeting a highly conserved region of the matrix (M) gene located between nucleotide positions 1911 and 2261 derived from the alignment of sequences published in GenBank [5] was used to screen for the presence of ABV sequences from all samples. (Forward, 5'- GGTAATTGTTCCTGGATGG-3' (Positions 1911–1930). Reverse 5'- ACACCAATGTTCCGAAGACG-3' (Positions 2261–2242).

PCR conditions were: initial denaturation, 94°C for 2 min, followed by 35 cycles of 94°C for 30 sec, 55°C for 30 sec, and 72°C for 30 sec, followed by a final extension at 72°C for 5 min. RT-PCR assays included multiple reagent controls to which no cDNA was added. Products of appropriate size were submitted to the Gene Technology Laboratory at Texas A&M University for Sanger sequencing.

### Genome cloning

Following growth in duck embryo fibroblasts for 2 passages, cDNA was amplified from the DEFs as described above. Two sets of primers (5'- TGTTGCGTTAACAACAAACC-3' (Positions 1–20, corresponding to the 3’ terminus), 5'-ACACCAATGTTCCGAAGACG-3' (Positions 2261–2242) and 5'- GGTAATTGTTCCTGGATGG-3' (Positions 1911–1930, 5'-TGCGCTACAACAAAGCAACAACC-3' (Positions 8914–8892, corresponding to the 5’ terminus) were used to amplify the viral genome by generating two products, approximately 3 and 6 kb in size. The PCR conditions were an initial denaturation, 94°C for 5 min, followed by 35 cycles of 94°C for 1 min, 55°C for 1 min, and 72°C for 7 min, followed by a final extension at 72°C for 5 min. PCR products were cloned into the pCRTM4-TOPO vector. They were then sequenced using a primer-walking approach by the Gene Technology Laboratory of Texas A&M University. Complete viral sequences were assembled using Sequencher 4.1, and phylogenetic trees based on the partial M gene and complete genome without the 3’ and 5’ termini were constructed using the Neighbor-Joining tree build method with the Jukes-Cantor model and 1000 bootstrap replicates by MEGA 6.0.

The genome sequences obtained were submitted to Genbank. The accession number is GenBank: KJ756399.

### Histopathology

Brains and eyes were removed from all 13 ABV-positive birds and two negative birds. Brains were sectioned along the mid-line and one half of the brain and one eye from selected birds were fixed in 10% neutral buffered formalin. These tissues were trimmed, processed, sectioned and stained with haematoxylin and eosin.

### Immunohistochemistry (IHC)

Selected fixed brain and eye tissue samples were examined by immunohistochemistry using a polyclonal antiserum directed against ABV nucleoprotein according to the modified procedure of Wünschmann et al. [12]. The polyclonal antiserum was raised in rabbits against the cloned nucleoprotein from ABV genotype 3 and was kindly provided by Drs. Ian Lipkin and Thomas Briese.

## Results

### Detection of ABV in ducks

Twelve of 83 mallard brains and one of 8 wood duck brains subjected to reverse transcriptase-PCR generated a PCR product of 350 bp, the expected size. No product was detected in four green-winged teal and three pintail brains. Sequencing provided 174 nt of high quality data and confirmed that ABV of a single genotype was present in all positive cases and that its M gene sequence was different from previously reported ABV genotypes. The PCR product demonstrated only 79% sequence identity to its closest relative, the M gene of the ABV-CG genotype. Partial M gene sequences obtained from 12 of the 13 positive brains were identical. The partial M gene sequence obtained from Mallard 77 showed 97% identity with the others (See Additional file 1).

### ABV isolation

In order to confirm the identity and presence of this ABV in brains, 2 positive brain samples were used to inoculate primary duck embryo fibroblast cultures. The virus grew readily and was detected by RT-PCR at 14 days from both brain inocula. The persistently infected DEFs were successfully passaged 10 times.

### ABV genotyping

RT-PCR products from a single cultured virus (MALL-89) at passage 2 were cloned into pCR™4-TOPO vector and genome sequences were determined. The sequenced component of the genome of this ABV comprised 8904 nucleotides organized in a manner very similar to that observed in other ABV genotypes (Data not shown). When compared to genotype CG, nucleotide identities ranged from 84% in the N gene to 72% in the L gene. Amino acid sequence identities ranged from 95% in the N protein to 79% in the X protein. Average nucleotide identity across the genome was 72% (Table 1).

**Table 1:** **A comparison of nucleotide and amino acid identity between the two waterfowl isolates of Avian Bornavirus, ABV-MALL and ABV-CG**

	**NT identity% (length)**	**AA Identity % (length)**
N	77 (1116)	95 (371)
X	84 (264)	79 (87)
P	79 (609)	87 (202)
M	80 (429)	94 (142)
G	74 (1500)	80 (499)
L	72 (6439)	81 (1714)
Overall	72 (8950)	83 (3015)

**Figure 1:**
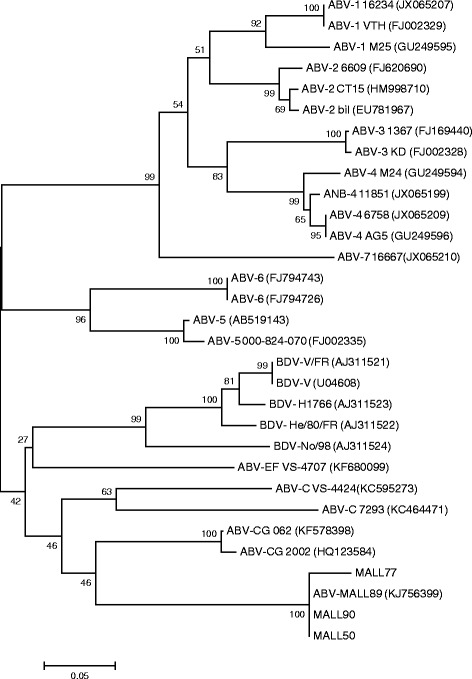
**Unrooted phylogenetic tree constructed from partial M gene sequences using the Neighbor-Joining tree build method with no outgroup and 1000 bootstrap replicates using MEGA 6.0.**

**Figure 2:**
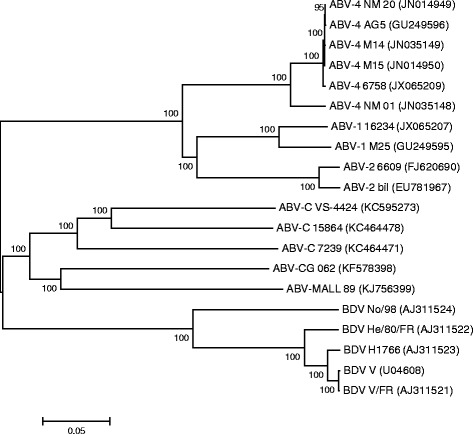
**Unrooted phylogenetic tree constructed from published complete ABV genome sequences using the Neighbor-Joining tree build method with no outgroup and 1000 bootstrap replicates using MEGA 6.0.**

Phylogenetic analysis based on the partial M gene sequences, indicated that the mallard virus genotype is distinctly different from ABV-CG, its closest relative (Figure 1). A similar pattern was obtained from the phylogenetic analysis based on published complete ABV genome sequences (Figure 2).

### Clinical and pathological lesions

Enquiries to the local wildlife personnel and the hunters involved in sample collection, failed to reveal any evidence of disease or unusual mortality events among duck flocks in the refuge. Nevertheless, based on previous observations in ABV-infected geese, we considered it possible that the birds, while clinically “normal” could be suffering from a subclinical viral encephalitis.

**Figure 3:**
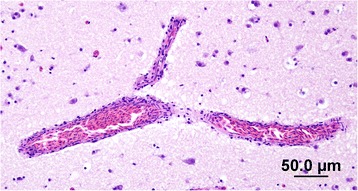
**Section of the brain of an ABV-infected mallard showing minimal perivascular cuffing.** H&E stain.

**Figure 4:**
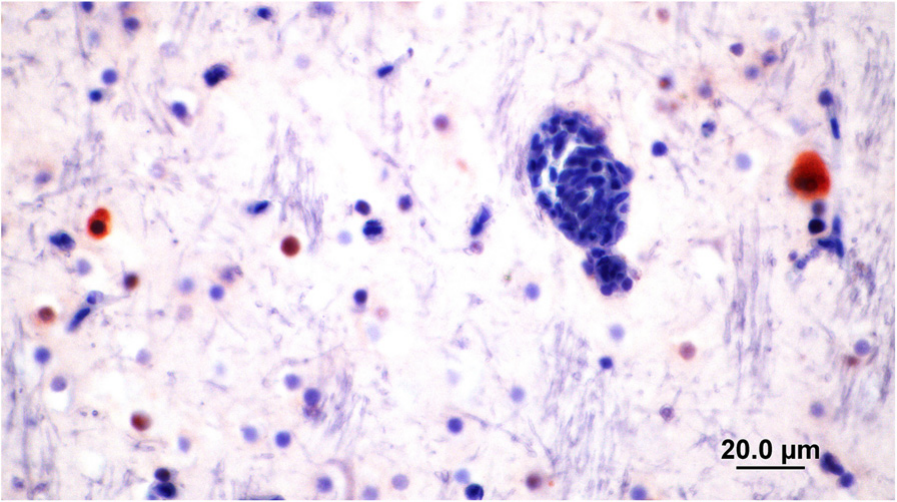
**Section of mallard brain stained with rabbit anti ABV-N showing ABV nucleoprotein in the nucleus and cytoplasm of glial cells.**

**Figure 5:**
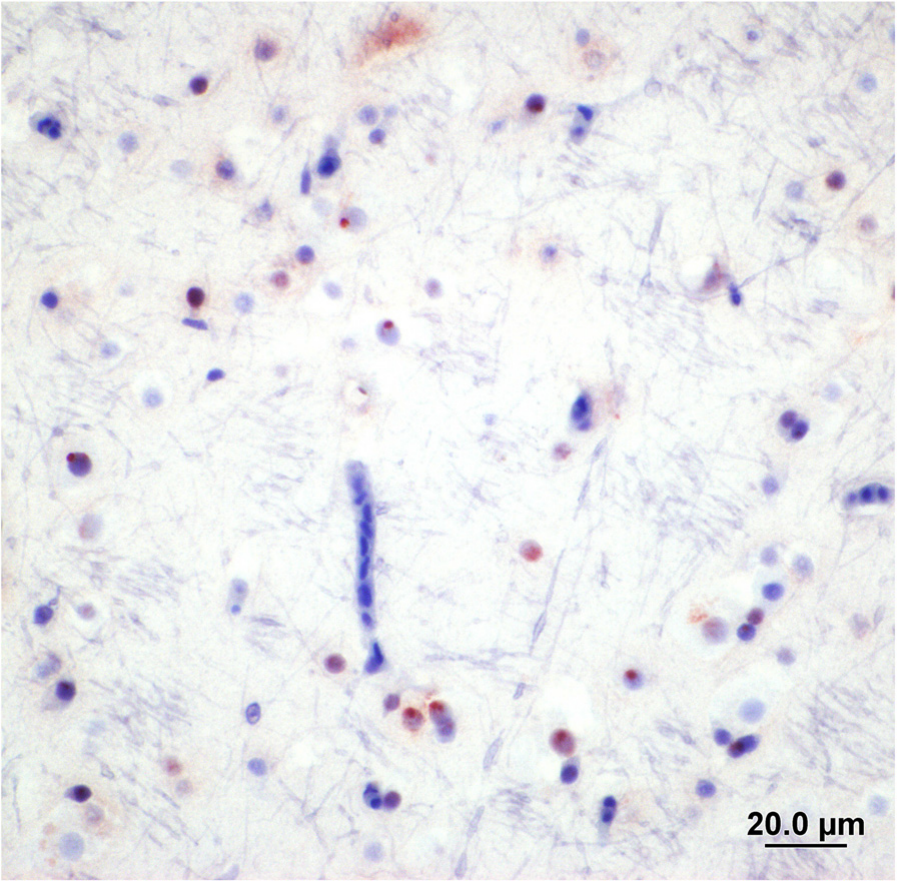
**Section of mallard brain showing the presence of viral N protein mostly in the nuclei of glial cells.** The nuclear staining is typical of that expected from a bornavirus.

**Figure 6:**
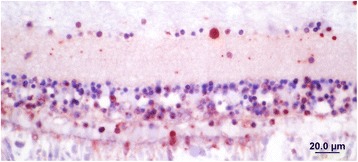
**Section of mallard retina showing the presence of ABV nucleoprotein in the nucleus and cytoplasm of cells in the ganglion and nuclear cell layers as well as in some rods and cones.**

Although brain and eye tissues from all 13 positive ducks and two negative ducks were processed for histopathology and immunohistochemistry only four were suitable for examination due to freezing artifacts. Histopathological examination of the four PCR-positive brains showed minimal signs of encephalitis such as mild perivascular cuffing and increased glial cell numbers in the cerebrums but not in the eyes (Figure 3). However IHC of the brains that had not been frozen revealed the presence of ABV-positive cells in both the brain (Figures 4 and 5) and the eyes of PCR-positive birds (Figure 6). The ABV nucleoprotein was present in the nucleus and cytoplasm of mainly glial cells and a few neurons within the brain. The intense staining observed in the nuclei (Figure 5) is consistent with bornavirus infection. Similarly, in the infected retina, ABV nucleoprotein was detected in both nucleus and cytoplasm of scattered cells within the ganglia, inner and outer plexiform layers and in rods and cones (Figure 6). A few scattered glial cells within the optic nerve also contained ABV nucleoprotein.

## Discussion

The site studied in this report, Eufaula Lake lies in the center of the North American continent. Waterfowl in North America use several different routes (flyways) to travel between their wintering grounds in the south to their nesting grounds further north. Eufaula Lake lies in the midst of the central flyway. Thus mallards that winter at this site nest in the northern Canadian prairies, most notably in Saskatchewan and Manitoba. The presence of such a high prevalence of infection at a wintering site suggests that a broader survey of nesting birds in the northern Canadian prairies may well reveal the presence of additional infected populations and possibly new ABV genotypes. The primer pair used to detect the presence of ABV was derived from sequences previously published for the Canada goose genotype in Genbank. The primer sequences were not completely identical to that in the mallard. It is possible therefore that primers based on this new genotype may have detected more infected birds in this flock.

This new genotype of ABV has 72% overall sequence identity with the Canada goose genotype (ABV-CG), its closest relative [13] (Figure 1). We have therefore tentatively named it the Mallard genotype (ABV-MALL). The differences between these two genotypes is of a similar magnitude to that previously reported between the different psittacine genotypes [14] and reflects the great diversity of this family of viruses.

The detection of at least 13 distinct genotypes of ABV to date, stands in marked contrast to the very limited diversity observed in mammalian Borna disease virus where only two genotypes have been detected [14]. The ABV genotypes do differ on the basis of epidemiology and geography. Thus currently circulating strains of BDV appear to have been largely restricted to a relatively small area of central Europe. They have a specific reservoir host in the shrew, *Crocidura leucodon* [15] and possibly also bank voles (*Myodes glareolus*) [16]. This is in marked contrast to ABV-CG that is found across the North American continent and where no mammalian reservoir host has been identified [7]. Likewise the worldwide distribution of the psittacine ABV genotypes probably reflects the extent of the international trade in captive parrots rather than the situation in wild parrot populations. Since the differences between BDV and ABV are no greater than those between other ABV genotypes, it may be appropriate to consider BDV to be simply another ABV genotype [14].

A characteristic feature of all ABV genotypes is their unpredictable pathogenicity. Thus in parrots and waterfowl, infection is relatively common but disease is rare. For example, 14% of Canada geese across North America are healthy carriers of ABV [5]. Diseased birds have been identified in a flock in Toronto involving 30/132 birds and in an additional 21/823 birds in Ontario [10]. Delnatte’s studies on waterfowl with neurologic disease in Ontario suggested that ABV is the major cause of encephalitis in these species [17]. Delnatte and her colleagues have reported a diversity of clinical signs including not only gastrointestinal impaction, but also behavioral abnormalities, and severe disabilities including an inability to walk or fly. It has been argued that ABV is the major infectious cause of neurologic disease in wild geese and swans in Ontario [10]. Cases of what appears to be classical PDD have also been reported from Canada geese in Prince Edward Island association with ABV-CG infection [11]. ABV neurologic syndrome has also been reported in an overwintering flock of Canada geese in Massachussetts (M. Murray, Personal communication) and in a few Canada geese in California (Shivaprasad, Unpublished).

The presence of virus in the eyes of infected mallards may be significant. Severe inflammation leading to blindness is a feature of Borna disease in mammals [18,19]. It develops in naturally infected horses and is a feature of Borna disease induced in adult rats. In the case of rats, Borna virus replicates in the nervous system with the greatest concentration of virus in the cerebrum and eyes. This is accompanied by microglial infiltration in the ganglion cell and inner plexiform layers. Blindness is not a common feature of PDD in psittacines [20] although a lymphocytic choroiditis is occasionally observed (Shivaprasad, unpublished). In the case of the mallards studied here, minimal cellular infiltration of the retina was observed and there was no evidence of retinitis. Nevertheless IHC indicated that neurons in the ganglion cell layer, the nuclear cell layers and the photoreceptors were infected with ABV. There were some rods and cones infected as well. Thus it is possible that interference with neural transmission could affect their vision. This, while not immediately lethal may well reduce their survival in the wild.

Borna disease virus has been shown to cause disease in laboratory rodents as a result of T-cell mediated cytotoxicity [21]. It is not known whether this is the case in birds infected with ABV. We suggest that the intensity of their T cell response may determine whether a bird develops clinical neurologic disease. This may be a function of age at first exposure. Thus young birds infected from their mother *in ovo* may be tolerant to the virus. ABV can be readily detected in embryos, although there have been no reports of viral isolation [22,23]. Given that ABV is noncytopathic, a persistent infection may therefore fail to generate strong T cell immune responses resulting in inflammation. On the other hands, birds infected for the first time as adults may recognize the virus as foreign and thus trigger a T cell response leading to the development of encephalitis and clinical disease.

## Conclusions

Avian bornaviruses appear to be relatively common in some wild bird populations. ABV of the Canada goose genotype is not the only Avian Bornavirus genotype circulating in wild waterfowl in North America. A new genotype, provisionally designated ABV-MALL was present in a population of wild mallards and a wood duck sampled in Oklahoma. Given the migratory nature of these birds, it is likely to be widely distributed across the central United States and Canada. Since this genotype was isolated from apparently healthy birds, its significance as a pathogen is unclear. Other ABV genotypes however, have been associated with neurologic disease in wild waterfowl so this genotype may be potentially pathogenic. In this specific case, infected ducks appeared to be healthy enough to remain with their flocks. It is also possible, given the presence of virus-infected cells in their retinas, that the birds may have been visually impaired. While not lethal this could adversely affect their survival in the wild.

## Additional file

Additional file 1:
**Mallard M gene sequences.**
