# Supplementary material for: Characterization of a new genotype of avian bornavirus from wild ducks
Source: Virol J. 2014 Nov 19;11:197. doi: 10.1186/s12985-014-0197-9 (PMC4239314; doi:10.1186/s12985-014-0197-9)
Supplement: Additional file 1: — Mallard M gene sequences. [file 12985_2014_197_MOESM1_ESM.pdf]

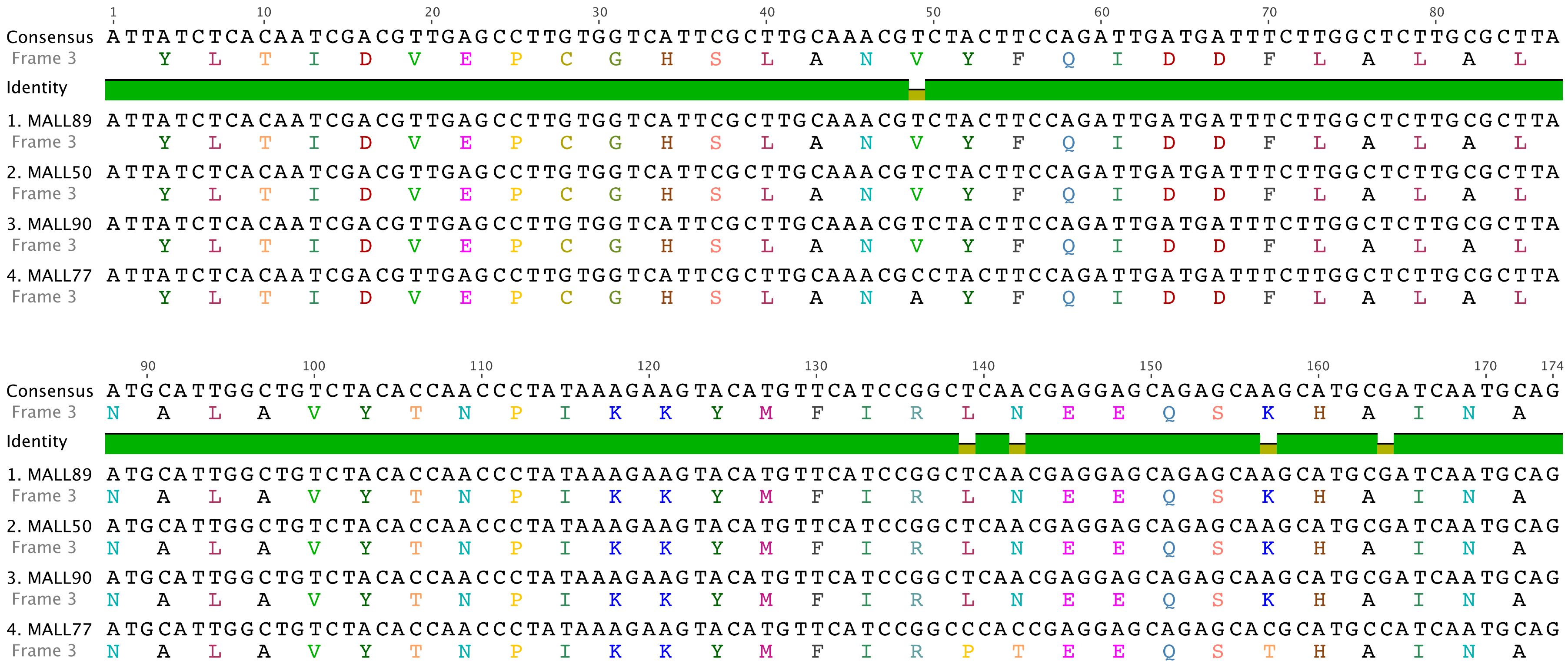
1 10 20 30 40 50 60 70 80
Consensus ATTATCTCACAATCGACGTTGAGCCTTGTGGTCATTCGCTTGCAAACGTCTACTTCCAGATTGATGATTTCTTGGCTCTTGCGCTTA
Frame 3 Y L T I D V E P C G H S L A N V Y F Q I D D F L A L A L
Identity
1. MALL89 ATTATCTCACAATCGACGTTGAGCCTTGTGGTCATTCGCTTGCAAACGTCTACTTCCAGATTGATGATTTCTTGGCTCTTGCGCTTA
Frame 3 Y L T I D V E P C G H S L A N V Y F Q I D D F L A L A L
2. MALL50 ATTATCTCACAATCGACGTTGAGCCTTGTGGTCATTCGCTTGCAAACGTCTACTTCCAGATTGATGATTTCTTGGCTCTTGCGCTTA
Frame 3 Y L T I D V E P C G H S L A N V Y F Q I D D F L A L A L
3. MALL90 ATTATCTCACAATCGACGTTGAGCCTTGTGGTCATTCGCTTGCAAACGTCTACTTCCAGATTGATGATTTCTTGGCTCTTGCGCTTA
Frame 3 Y L T I D V E P C G H S L A N V Y F Q I D D F L A L A L
4. MALL77 ATTATCTCACAATCGACGTTGAGCCTTGTGGTCATTCGCTTGCAAACGCCTACTTCCAGATTGATGATTTCTTGGCTCTTGCGCTTA
Frame 3 Y L T I D V E P C G H S L A N A Y F Q I D D F L A L A L
90 100 110 120 130 140 150 160 170 174
Consensus ATGCATTGGCTGTCTACACCAACCCTATAAAGAAGTACATGTTCATCCGGCTCAACGAGGAGCAGAGCAAGCATGCGATCAATGCAG
Frame 3 N A L A V Y T N P I K K Y M F I R L N E E Q S K H A I N A
Identity
1. MALL89 ATGCATTGGCTGTCTACACCAACCCTATAAAGAAGTACATGTTCATCCGGCTCAACGAGGAGCAGAGCAAGCATGCGATCAATGCAG
Frame 3 N A L A V Y T N P I K K Y M F I R L N E E Q S K H A I N A
2. MALL50 ATGCATTGGCTGTCTACACCAACCCTATAAAGAAGTACATGTTCATCCGGCTCAACGAGGAGCAGAGCAAGCATGCGATCAATGCAG
Frame 3 N A L A V Y T N P I K K Y M F I R L N E E Q S K H A I N A
3. MALL90 ATGCATTGGCTGTCTACACCAACCCTATAAAGAAGTACATGTTCATCCGGCTCAACGAGGAGCAGAGCAAGCATGCGATCAATGCAG
Frame 3 N A L A V Y T N P I K K Y M F I R L N E E Q S K H A I N A
4. MALL77 ATGCATTGGCTGTCTACACCAACCCTATAAAGAAGTACATGTTCATCCGGCCCACCGAGGAGCAGAGCACGCATGCCATCAATGCAG
Frame 3 N A L A V Y T N P I K K Y M F I R P T E E Q S T H A I N A
